# Assessment of evidence from teeth and the alveolar bone of skulls

**DOI:** 10.1038/s41598-025-23880-5

**Published:** 2025-11-17

**Authors:** Scheila Mânica, Julieta Gómez García-Donas, Tobias Houlton, Hemlata Pandey

**Affiliations:** 1 Centre for Forensic and Legal Medicine and Dentistry, School of Dentistry, University of Dundee, 2 Park Place, Dundee, DD1 4HN Scotland, UK; 2 Centre for Anatomy and Human Identification, Medical Science Institute, Dow Street, University of Dundee, Dundee, DD1 5EH UK

**Keywords:** Forensic odontology, Dental anthropology, Anthropology, Tooth loss, Tooth wear, Anatomy, Diseases, Health care, Medical research

## Abstract

A notable lack of agreement on a standardised criterion for representing changes in alveolar bone persists, which limits its application in forensic dentistry. This study aimed to describe the data on loss of tooth substance and alveolar bone in skulls. Dental and trabecular bone information from 28 skulls from the German Helmer skull collection was collected through dental charting, intraoral photographs taken with a DSLR camera (Nikon D5600, Nikon Corporation, Japan), and small radiographs obtained using a handheld dental X-ray device (Nomad Pro 2, Kavo Kerr, Biberach, Germany). Adobe Photoshop 26.4.1 was used to create diagrammatic representations of alveolar socket changes following tooth loss. Blunt force skull trauma affected tooth insertion in two cases. Most of the dental trauma involved enamel-dentine (n = 48; 74%), and untreated decay was relatively low (n = 14). Most missing teeth were lost *antemortem* (n = 283; 87%). Dehiscence was the most prevalent pathology seen in the trabecular bone (n = 26), followed by fenestration (n = 14). While fracture propagation patterns and tissue changes provide preliminary diagnostic criteria for assessing the timing of tooth or bone fracture, the assessment of events *perimortem* continues to challenge. The summary of alveolar socket changes could be of assistance in legal reports and teaching.

## Introduction

The use of archaeological and contemporary skull collections highlights the intersection of dentistry and anthropology. Studies included the assessment of caries in ancient and modern populations^1^, non-metric dental traits and craniometric analyses for biogeographical origin^2^, early experiences in dental pathology^3^ and the significance of teeth in understanding human evolution and identity in both living and ancient dental remains^4^. Those studies illustrate the multifaceted contributions of dental professionals to archaeological and anthropological research. It is also important to note the importance of forensic dentists assessing skull collections to lead morphological and morphometric investigations of anatomical dental traits, dental treatments and dental materials^2,5,6^.

In comparative dental analysis, expected, available and comprehensive *antemortem* (before death) records are compared with *postmortem* (after death) dental findings^7–10^. The dental assessment uses morphological, therapeutic (dental treatment), and pathological dental features, and even non-dental features in the jaws, such as the shape of the maxillary sinus and inferior alveolar canal and alveolar bone morphology (bone surrounding and supporting the teeth) due to congenital tooth shape and alignment^11–13^. In cases where teeth are missing *postmortem*, intra-alveolar impressions can provide information about root morphology using reconstructive techniques to capture intra-alveolar morphology such as the use of dental impression materials^14^ or radiopaque mixtures into sealed sockets for radiographic comparison^15^. When *antemortem* data is not available, dental profiling is performed to gather evidentiary information through the study of teeth and oral tissues, including age, sex, biogeographical origin, personal habits and diet^16^.

A clear and coherent expert evidence presentation is a shared responsibility between forensic scientists and legal professionals^17^. Improving cross-disciplinary communication requires professional development initiatives, and forensic experts must effectively convey complex scientific concepts to diverse audiences within the constraints of the criminal justice system^18^. In forensic dentistry, questions about tooth extraction are often posed in two situations: (1) during comparative dental analysis where teeth are missing in the human body or remains but present in the *antemortem* dental records; and (2) in cases of dental age estimation using third molars, particularly for determining whether an individual has reached the legal age of 18^19^. Explaining scientific and clinical terms related to missing teeth, including possible causes and the timeline, can be challenging. The aim of this study was twofold: first, to describe the data involving loss of tooth substance and alveolar bone in skulls from the Helmer collection; second, to didactically present discriminatory representation of bone features (and also soft tissues) of *ante- or postmortem* dental sockets after tooth loss.

## Teeth missing *antemortem, perimortem* and *postmortem,* and effects on the alveolar bone

Understanding when the tooth loss occurred and the underlying causes can be challenging. *Antemortem* tooth loss occurs during the individual’s lifetime due to pathological factors such as caries, pulpitis, or periodontitis, trauma (accident or usage) or extraction for therapeutic purposes^4,20^. The alveolar bone forms the walls of the dental socket, which is internally lined by the lamina dura. The sockets of teeth missing *antemortem* (MAM) often present evidence of healing, such as smooth socket walls and alveolar bone remodelling, which can be detected radiographically^21^. The healing process involves the modelling and remodelling of both hard and soft tissues, including alveolar bone, periodontal ligament, and gingiva, with granulation tissue replaced by a provisional matrix and woven bone between 2 and 8 weeks^22,23^. Various factors can influence socket healing, and the cumulative effect of changes in the alveolar ridge includes local changes to the composition of the compact and cancellous bone, a reduction in local bone density and alteration to the height, width and three-dimensional morphology of the site^21,24^.

*Postmortem* tooth loss is often influenced by environmental factors such as scavenging, decomposition, *postmortem* interval, improper handling of remains, excavation methods, root morphology, and advanced age at the time of death^25–27^. The sockets of teeth missing *postmortem* (MPM) typically present as sharp, unhealed edges and may hold debris^14^. Radiographically, there is no sign of bone healing. The conical shape of anterior single-rooted teeth is a well-documented contributing factor because of the reduced surface area and limited mechanical retention after the loss of periodontal ligament attachment compared to multi-rooted posterior teeth^28^. Moreover, short roots and increased root taper at specific teeth could be considered as risk indicators for periodontitis, which would worsen the susceptibility of anterior teeth to *postmortem* loss^29^. A study found a high prevalence of maxillary incisor loss *postmortem*, possibly due to exposure of the cranium or the weaker bone density of the maxilla compared to the mandible^30^. The environment influences the taphonomy: during summer months, the loss of teeth *postmortem* is much more rapid than during the late fall or winter months. Similarly, the deceased exposed to direct sunlight tend to lose teeth more easily due to the micro-environment where rapid decomposition occurs as opposed to shaded locales^31^.

In a discrimination between *perimortem* and *postmortem* tooth loss, there is no display of evidence of remodelling or resorption of the alveoli of teeth lost within days to a week of the death^32^. *Perimortem* tooth loss occurs around the time of death, when the tissues are still hydrated, and thus react differently to trauma^33^. It must result from decay or trauma rather than the shifting and settling of the skeleton after death. *Postmortem* tooth loss may occur because teeth might detach from the jawbone due to the decomposition of the periodontal ligament (PDL), the fibrous tissue that anchors teeth to the alveolar bone^34^. A three-dimensional (3D) digital model named *‘Dental damage 1’* represents the bone of tooth loss in the region of the lower left second molar (tooth#37) and the bone of postmortem tooth loss in the region of the lower left third molar (tooth#38)^35^. A recent study found that the relationship between the mesiodistal and buccolingual alveolar diameters from skulls can be used to provide information about missing teeth^36^.

## Dental pathologies

Dental pathologies affecting bone are diverse and can stem from systemic disorders or local factors. Common conditions include periodontal disease and endodontic lesions, both involving bacterial-induced inflammation leading to bone resorption and the presence of calculus^37^. Socket morphology can reveal evidence of periodontal disease, infections or other conditions that led to tooth loss, providing insights into the individual’s oral health^4^. Rare bone diseases, pediatric bone disorders such as fibrous dysplasia, Paget’s disease, osteogenesis imperfecta and antiresorptive therapy in cancer patients can have significant maxillofacial manifestations^38–40^. Lesions such as cysts are found exclusively in the jaws due to their intimate relationship to the teeth. Odontogenic cysts, including primordial, dentigerous and radicular cysts fluid-filled sacs that arise from the epithelium associated with tooth development, classified as either developmental or inflammatory and odontogenic tumours. Non-odontogenic cysts arise from tissue not involved in tooth formation, and non-odontogenic tumours^41,42^.

The study of caries in archaeological collections focuses on differences in caries and dietary patterns in different populations. The assessment include the great number of teeth lost *postmortem* and the severe tooth wear that exposes and infects the pulp cavity, tooth root and resorption of the alveolar bone, which affects the tooth support. It is noted that if wear is extreme, the abscess is more likely to be due to attrition rather than caries, although it may not always be possible to discriminate^41^. A chronic dental abscess (a collection of pus surrounded by denser tissue) may appear at the tip of the root or in association with general periodontal infection^43^.

Alveolar bone resorption is a characteristic of periodontitis, and the distance between the cementoenamel junction (CEJ) and the alveolar crest (AC) is a common quantitative parameter used, but the palatine exposure of the root caused by ageing or occlusal wear may be mistakenly interpreted as evidence of periodontitis^44^. Moreover, other alveolar bone defects can be seen, such as fenestration (absence of part of the lingual or buccal alveolar bone lamina and root exposure) and dehiscence (an increase in the distance between the cementoenamel junction and the alveolar bone crest), which are usually observed more in the facial than lingual root surfaces. The aetiology includes the tooth (position, curvature of the root), occlusion (trauma, force, movement) and the thickness of the alveolar bone. Both defects are prevalent in the upper and lower incisors, upper canines, upper premolars, and upper molars^4,43,45,46^.

### Dental trauma

Analysis of dental and maxillofacial skeletal trauma is crucial in both living and deceased individuals, particularly in cases of suspected abuse, causing traumas, including high-velocity projectiles, sharp or blunt force, and thermal injuries^47,48^. The expertise in biomechanics should elucidate the nature of trauma, the mechanism of insult, and the impact forces that create traumatic lesions, with forensic odontologists playing a vital role in examining dental injuries^49–51^.

There appears to be a relationship between impact location and the location of fracture in cases involving a single mandibular fracture; other predisposing factors include systemic or local diseases (tumors, osteoporosis, Paget’s disease, histiocytosis X, and multiple myeloma), weaknesses of the mandible caused by abscesses, *antemortem* tooth loss and the presence of impacted or unerupted teeth such as third molar teeth and maxillary canine teeth, including its angulation^52–54^. A study on condyle fractures found that the lack of occlusal support is more associated with condyle fractures than the presence of occlusal support, regardless of third molar presence and characteristics and other variables evaluated^55^. Dentoalveolar trauma affecting teeth, periodontium, and alveolar bone accounts for approximately 15% of emergency room visits, and frequent etiological factors in adults include traffic accidents and physical assault^56–59^. Examples of such injuries include the avulsion of teeth, fractures of the teeth and fractures of the alveolar process^57^ including avulsion and coronal/crown-root fractures, the most common types of dentoalveolar injuries^58^. Usual complications of dentoalveolar trauma are pulp necrosis, pulp canal obliteration, periapical pathosis and root resorption^60^.

It is challenging to differentiate *antemortem* and *perimortem* trauma from *postmortem* damage in dry bone specimens. The literature on these distinctions in bone is abundant, as opposed to human dentition^61^. For the prior, the assessment of the timing of injuries is dependent upon the evidence of their healing and remodelling, or the lack thereof^61^. Frequently, *postmortem* damage involves the enamel, which chips off easily, but major portions of the tooth, including the dentin and pulp cavity, may split. The smoothing of the fractured edges of the tooth is a possible criterion for making the distinction between *ante*- and *postmortem* fractures, which would occur in *antemortem* fractures if sufficient time had elapsed between the fracture and death^41^. Pulp canal obliteration is a pulpal response to trauma, characterised by the deposition of hard tissue in the root canal lumen and could be seen in such cases^62^. The exposed dentine can also present a yellow and brownish colour^41^.

## Materials and methods

Two forensic dentists (SM and HP) analysed 28 skulls of German citizens (20 males; 7 females; 1 unknown), aged 30–80 years, from the Helmer skull collection. Prof Dr Richard Helmer collected this material from forensic investigations, a combination of cold cases and unclaimed identified, largely from the 1970s, which was donated to the Centre for Anatomy and Human Identification, University of Dundee, UK, for academic and research purposes since 2005^63,64^. Interestingly, there is a connection between Dr Richard Helmer and forensic odontology, which happened during the identification of Josef Mengele’s remains in the 1980s. Mengele was a Nazi SS officer and physician at Auschwitz, infamous for his inhumane medical experiments on prisoners. Dr Helmer used craniofacial superimposition of Mengele’s skull^65^, exhumated in Brazil in 1985, whose findings added to other experts that examined the teeth^66^, and also performed the DNA analysis^67^.

The collection of dental data was a three-step procedure: (1) photographs of the skulls’ maxilla and mandible were taken in three views: frontal, two laterals (right and left hand side) and occlusal of upper and lower dentition using a DLSR camera (Nikon D5600, Nikon Corporation, Japan); (2) dental status was charted using clinical instruments (stainless steel small mirror and exploratory probe); and (3) small radiographs were taken using a handheld dental X-ray device (Nomad Pro 2, Kavo Kerr, Biberach, Germany). Anthropologists previously established information about sex, age and skull trauma, but a further assessment of fractures only impacting teeth was performed (JGGD).

Dental data was categorized as (a) missing *antemortem* (n); (b) missing *postmortem* (n); (c) decay untreated (interproximal crown, lateral (buccal) crown, occlusal; root; non-existent); (d) fracture (only enamel; enamel and dentine; pulp exposure); (e) wear (only enamel; enamel/ dentine; pulp exposure) and (f) non-specific bone pathology/anomaly (abscess; dehiscence; cysts; fenestration) as seen in Table 1. Following, the healing stages of alveolar bone following *antemortem* tooth loss *(*stages 1–4) along with a stage standing for *postmortem* tooth Loss (stage 5) were represented by photographs and radiographs derived from the skulls (stages 2–5), except for stage 1, the images are clinical (courtesy of Dr. Vinny Karia, Oak Ridge Dental, California). Adobe Photoshop 26.4.1 was used for diagrammatic representations and descriptions of skull assessments and previous studies^21,22^.

**Table 1:** Overall dental data distribution from the skulls according to all categories.

Skull code (case)	Sex*	Age (Y)	Teeth missing AM	Teeth missing PM	Tooth decay untreated**	Tooth fracture***	Tooth wear****	Non-specific bone pathology/anomaly*****
1	F	30–40	1 (LP)	0	N	0	E, n = 2 (P) D, n = 9 (6 A & 3P)	F, n = 2 (UP)
2	M	30–40	2 (LP)	2 (LA)			E, n = 10 (P) D, n = 5 (4 A & 1P)	A, n = 2 (UA); D, n = 10 (6 LA & 4 LP)
3	F	15–20	0	1 (UP)		D, n = 2 (A)	0	F, n = 1 (UP)
4	M	50–60	6 (UP)	2 (LP)		P, n = 1 (A)	D = 2 (A)	0
5	F	30–50	1 (LP)	0		D = 4 (3 UA & 1LA)	D = 4 (A)	F, n = 2 (UP)
6	M	30–40	12 (2 LA, 5 LP & 5 UP)	7 (1 LA, 1 UP & 5 LP)		0	E = 2 (A&P)	A, n = 1 (UA)
7	M	35–45	20 (6UA, 2LA, 6UP & 6LP)	0			E, n = 4 (P) D, n = 1 (P)	D, n = 2 (1UA & 1 UP)
8	M	40–50	27 (6UA, 2 LA, 10UP & 9LP)				D, n = 5 (3A & 2P)	D, n = 5 (3LA & 2LP)
9	M	40–60	3 (LP)			D, n = 3 (A)	D, n = 8 (A)	0
10	F	–	6 (4UP & 3LP)		OI, n = 1 (P)	0	E, n = 7 (P); D, n = 9 (A)	D, n = 1 (UA)
11	F	–	4 (2UP & 2LP)		O, n = 1 (P) OI, n = 1 (P)	P, n = 1 (A)	E, n = 3 (P)	0
12	F	–	11 (1UA & 5UP, 5LP)		N	0	E, n = 5 (P)	F, n = 2 (UA)
13	U	–	28 (4UA, 8UP, 6LA, 10LP)				0	A, n = 1 (UA)
14	M	–	10 (8UP & 2LP)		OI, n = 2 (P) L, n = 6 (A)	D, n = 1 (A)	E, n = 6 (A)	A, n = 1 (UA) F, n = 2 (1UP, 1LP)
15	M	–	27 (4UA, 6LA, 9UP, 8LP)		N	P, n = 5 (2A, 3P)	0	D, n = 3 (2UA, 1UP) F, n = 3 (2UA, 1LP)
16	M	–	1 LP			D, n = 3 (1A & 2P); P, n = 3 (A)	E, n = 1 (P) D, n = 3 (A)	0
17	M	–	9 (1UA,4UP, 5LP)			P, n = 2 (A) D, n = 12 (8A & 4P)	0	
18	M	–	1LP	0		D, n = 2 (A)	E, n = 27 (10A, 17P)	
19	M	–	0	20 (6UA, 3UP, 6LA, 5LP)		0	E, n = 2 (P)	F, n = 1 (UP) D, n = 2 (UP)
20	M	–	1LP	0		D, n = 10 (5A, 5P)	0	0
21	M	–	15 (2UA, 5UP, 8LP)	6 (1UA, 4LA, 1LP)		E, n = 2 (P)	D, n = 3 (A) P, n = 4 (2A, 2P)	D, n = 2 (UA)
22	M	–	25 (6UA, 8UP, 11LP)	0	I, n = 1 (A)	D, n = 3 (2A, 1P)	E, n = 1 (A)	C, n = 1 (UA)
23	M	–	30 (6UA, 9UP, 6LA, 9LP)	2 UP	N	0	0	0
24	M	60–80	14 (1UA, 7UP, 6LP)	0	L, n = 2 (P)	D, n = 6 (4A, 2P); P, n = 2 (A)	D, n = 11 (9A, 2P)	
25	M	–	15 (3UA, 7UP, 5LP)	2LA	N	D, n = 2 (P)	D, n = 6 (A)	F, n = 1 (LA)
26	M	–	13 (5UA, 4UP, 4LP)	0		0	D, n = 2 (A)	0
27	M	–	4 (1UP, 3LP)				D, n = 1 (A)	
28	F	45–55	10 (5UP, 5LP)			P, n = 1 (P)	D, n = 11 (A)	D, n = 1 (UA)

*Sex: M (male), F (female) and U (unknown); **Tooth decay untreated: N (non-existent), I (interproximal crown), L (lateral or buccal crown), O (occlusal), R (root); ***Tooth fracture: E (only enamel), D (enamel/ dentine), P (pulp exposure); ****Tooth wear: E (only enamel), D (enamel/ dentine), P (pulp exposure); *****Tubercular bone pathology/anomaly: A (Absces*s*), C (cyst), D (dehiscence), F (fenestration). Teeth’s positions were classified according to the location in the maxilla or mandible: U (upper teeth) or L (lower teeth); and the location on the jaws: A (anterior teeth, such as incisors and canines) and P (posterior teeth, such as premolars and molars).

## Results

Blunt force traumas to the skull also inflicted tooth fractures in two individuals, cases 5 and 10. Case 5 showed a para-symphyseal oblique complete fracture on the left side of the mandible, located between the lateral incisor and the canine. Case 10 presented a highly fragmented cranium, and skull fragments were previously reassembled with some bone areas missing, posing difficulties in fully reconstructing the traumatic events. This individual showed at least one impact with a comminuted fracture located on the frontal bone, slightly posterior to the right frontal eminence, and multiple mid-facial fractures presenting Le Fort fracture patterns. Regarding the dental structures, a sagittal fracture on the anterior surface of the right maxilla was observed extending from near the zygomaticomaxillary suture to the alveolar ridge between the canine and first premolar. Overall, most of the dental trauma involved enamel-dentine (n = 48; 74%), followed by reaching the pulp (n = 15; 23%), and only enamel (n = 2; 3%). Findings were not associated with the sex or age of the victims because of the uneven sample. Considering tooth loss, most missing teeth were lost *antemortem* in order: lower posterior (n = 114) > upper posterior (n = 100) > upper anterior (n = 45) > lower anterior (n = 24), totalling 283 teeth (87%) in comparison to 42 teeth lost *postmortem* (13%): lower anterior (n = 15) > lower posterior (n = 13) > upper anterior = upper posterior (n = 7 each). The number of teeth showing untreated decay was low (n = 14). Tooth wear affected mainly enamel-dentine (n = 80, 71 anterior and 9 posterior teeth), followed by enamel only (n = 70, 18 anterior and 52 posterior teeth). Considering the pathologies present in the trabecular bone, dehiscence was the prevalent one (n = 26, lower anterior (n = 9) > upper anterior (n = 7) > lower posterior (n = 6) > upper posterior (n = 4) followed by fenestration (n = 14, upper posterior(n = 7) > upper anterior (n = 4) > lower posterior (n = 2) > lower anterior (n = 1). Data is shown in Table 1. The Stages of the alveolar bone following tooth loss are shown in Table 2.

**Table 2:**
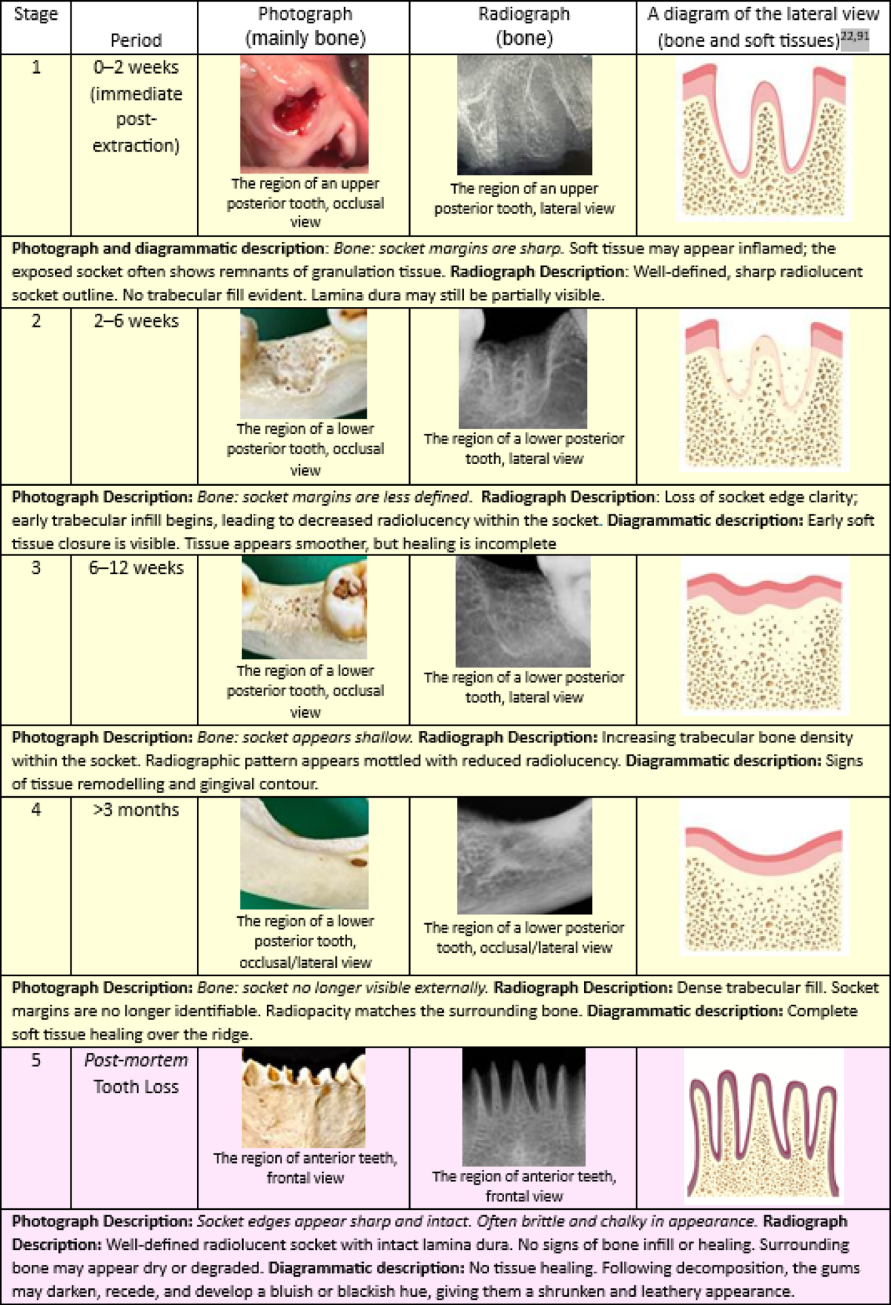
Summary of alveolar socket changes following tooth loss represented by photographic, radiographic and diagrammatic features^21,22^.

## Discussion

Even though it was not the scope of this study to assess skull fractures unrelated to teeth, most of the trauma cases were caused by blunt forces. These traumatically physical impacts can create depressed fractures with sharp, regular edges on the outer table and bevelled, irregular edges on the inner table^68^. It typically causes greater tissue deformation and vibration in the temporal bone compared to sharp injuries^69^. Concentric fractures can help differentiate between blunt force and ballistic trauma based on the bevelling direction^70^. In fatal head injury cases, blunt force is the most common cause of skull fractures, with comminated and linear fractures being the most prevalent types^71^. Research on blunt and sharp force trauma reveals varying patterns across different contexts. In the Russian Federation, fatal trauma rates from both blunt and sharp objects were significantly higher in some regions compared to national averages^72^. A study of conflict victims in Timor Leste found sharp force injuries to be most common (35%), followed by gunshot (20%) and blunt force (13.33%)^73^. However, in paediatric trauma cases in the United States, blunt trauma was far more prevalent (80.43%) than penetrating trauma (7.4%)^74^. Sharp force trauma can be categorised into stab wounds, incised wounds, chop wounds, and therapeutic/diagnostic wounds, with stab and incised wounds being the most common^75^. These findings highlight the importance of context in understanding trauma patterns.

Probable association between skull fractures affecting tooth positions were seen in two skulls of this collection. Facial fractures, particularly in the mandible, are often accompanied by dental injuries, with upper jaw teeth potentially at higher risk during mandibular fractures^76^. While the relationship between tooth and skull size shows low correlation in most skull types, brachycephalic individuals (short head) exhibit slightly higher correlations^77^. In this study, most dental trauma involved the enamel-dentine of anterior teeth. Enamel-dentine fractures are the most prevalent type of injury, followed by complicated crown fractures^78,79^. Maxillary central incisors are the most affected teeth, and males are more commonly affected than females^78–81^. Anterior teeth are also more susceptible to trauma and congenital defects^82^.

An earlier histological and histochemical investigation of undisturbed alveolar socket healing over 50 days offers valuable insight into the natural sequence of post-extraction repair. Alveolar socket healing following tooth extraction involves three overlapping phases: inflammatory, proliferative, and modelling/remodelling, as described by Amler^83^. The study reported that clot formation was the first response, which was subsequently replaced by granulation tissue by the 7th day. This was followed by the development of connective tissue around the 20th day. Interestingly, signs of osteoid formation appeared as early as the 7th day, and the socket was progressively filled with trabecular bone by approximately the 38th day. Additionally, epithelial coverage was seen as early as the 4th day, highlighting the rapid onset of mucosal healing. These findings are useful for understanding the biological timeline of socket healing in the absence of external interventions^83^. In forensic cases, this healing process can aid in estimating time since death, with initial stages of alveolar bone remodelling observable 13–42 days post-extraction^84^. *Postmortem* tooth loss is significantly affected by alveolar bone loss and strongly correlates with time since death^85^. Socket healing typically results in up to 50% reduction of the original ridge width, with greater bone resorption on the buccal aspect and in the molar region^86^.

The progression of alveolar socket healing following tooth loss is a well-orchestrated biological process, involving sequential histological and morphological changes that are observable both clinically and radiographically. Table 2 provides a structured overview of these changes, aiding in the differentiation between *antemortem, perimortem, and postmortem* tooth loss. In stages 1 through 4, the healing trajectory observed in *antemortem* cases aligns closely with established literature on socket repair. The inflammatory phase, typically occurring in the first week post-extraction, is characterised by clot formation and the appearance of granulation tissue^83,87^. The presence of a partially intact lamina dura and normal bone density during this stage further supports the classification of early *antemortem* loss.

As healing progresses into weeks 2–6 (Stage 2), granulation tissue is gradually replaced by connective tissue and immature bone. This period corresponds to the proliferative phase, during which fibroblast proliferation and early bone deposition occur^88^. Radiographically, the socket begins to show reduced clarity in its outline and early signs of trabecular infill, a critical marker of ongoing mineralization. These features are crucial in forensic settings to distinguish *antemortem* from *perimortem* or *postmortem* loss, particularly when evaluating the timeline of injury or trauma. Stage 3 (6–12 weeks) reveals further trabecular development and advanced soft tissue remodelling, with radiographs showing dense, mottled bone indicative of substantial healing. The gradual replacement of woven bone by more mature lamellar bone is observed during this phase^89^.

Stage 4, standing for healing beyond three months, shows a fully remodelled socket, with no visible socket margins either clinically or radiographically. This stage reflects the modelling/remodelling phase, where bone is reorganized to restore structural integrity and integrate with the surrounding alveolar bone^90^. The complete absence of the lamina dura and the return to homogeneous radiopacity are hallmarks of healed extraction sites. These morphological changes, coupled with gingival restoration, confirm long-standing antemortem healing and can serve as reliable indicators in forensic identification, especially when assessing older remains.

In contrast, Stage 5 presents unique features of postmortem tooth loss. The socket margins are sharply defined and dry, lacking any evidence of tissue healing or bone fill. The presence of an intact lamina dura and the absence of trabecular infill strongly suggest that tooth loss occurred after death. The bone may appear brittle and chalky, characteristics often seen in dry skeletal remains^91^. These distinctions are pivotal for forensic experts when determining the timing of tooth loss in unidentified individuals or trauma cases. While perimortem loss may sometimes mimic early antemortem loss, the absence of granulation tissue, lack of inflammatory response, and brittle bone quality in postmortem cases serve as clear differentiators.

Overall, the staged classification presented in Table 2 integrated photography, radiography, followed by interpretation, and diagrammatic representations. Mention should be made that all photographs (except Stage 1) are of bone, but the soft tissues are represented by the diagrams with further descriptions. Understandably, in dental autopsy, assessment of soft tissues is generally unnecessary, as decomposition or skeletonisation often eliminates these structures, leaving teeth and bones as the most reliable and enduring sources of information; however, the framework illustrated the soft tissues, which are mentioned in cases involving tooth extraction (*antemortem*) or even to represent the decomposition changes (*postmortem*). This study has not addressed the *postmortem* examinations of bodies that have suffered death by fire due to thermal damage and tissue destruction. Further research involving histological correlation and quantitative bone density measurements could strengthen these classifications and improve accuracy in determining the post-extraction timeline. A very short summary is presented in Table 3.

**Table 3:** A summary of the main discriminatory bone features of *ante-peri or postmortem* dental sockets after tooth loss.

Bone feature	Antemortem^21,24,112^	Perimortem^32,112^	Postmortem^14,31,32,91,112^
Socket edges	Rounded, remodelled	Sharp, minimal remodelling	Sharp, no remodelling
Lamina dura	Resorbed or remodelled	May be partially intact but disrupted	Fully intact
Surrounding bone	Increased radiopacity from remodelling	Similar density to fresh bone	No healing, possibly degraded
Bone healing	Trabecular bone fills the socket	No healing visible	No healing visible
Bone fractures	No fractures or healed fractures	Smooth or oblique fresh fractures	Jagged, dry, brittle fractures

According to the results of this study, most teeth lost *antemortem* were posterior teeth (molars and premolars). Posterior teeth, particularly mandibular molars, are more often extracted than anterior teeth due to a higher incidence of caries and periodontal problems^92^ Even though patients generally prefer tooth preservation over extraction when experiencing a toothache, socioeconomic factors influence this preference^93^. While dental caries remains the primary cause of tooth extraction across age groups, periodontal issues and orthodontic treatment also contribute to extraction patterns^92^. While caries affects posterior teeth more, periodontal disease tends to cause greater anterior tooth loss, especially in the lower jaw^94^. These findings highlight the complex interplay of factors influencing tooth extraction decisions. Another finding of this study is that most teeth lost *postmortem* were anterior teeth (incisors and canines). In living subjects, the loss of anterior teeth has a more significant impact on oral health-related quality of life compared to posterior tooth loss, affecting both physical and psychosocial functioning^95^. All data considered, it’s important to note that *postmortem* tooth loss can distort the proportions between anterior and posterior teeth in archaeological studies, potentially affecting caries prevalence calculations^96^. The question arises whether forensic diagnostic criteria can be developed to reliably distinguish the temporal origin of tooth fractures in medico-legal contexts. It has been observed that advanced imaging techniques consistently enhance the detection and localization of tooth fractures^97^. High-resolution computed tomography and cone beam computed tomography are cited for their improved accuracy in fracture visualization^98^. A study offering qualitative forensic criteria for temporal origin^33^ indicates that fractures propagating from enamel toward the dentin‐enamel junction suggest perimortem trauma, while those extending from internal dentin outward indicate postmortem change; another study^98^ notes that signs of calcification and bone resorption point to ante-mortem fractures, in contrast to the widespread cracking of postmortem specimens. Some studies focus on fracture detection, classification, or imaging guidelines without providing explicit temporal markers^99,100^. Thus, the reviewed literature supports only qualitative, unvalidated forensic indicators for distinguishing the temporal origin of tooth fractures in medico-legal contexts. Table 4 is a complementary table showing the main discriminatory features of dental trauma with no representative images.

**Table 4:** A summary of the main discriminatory dental features of *ante-peri or postmortem* tooth fractures.

Tooth fracture type	Healing signs^41,48,62,84^	Fracture appearance^41,48,62,84^	Radiographic appearance^41,48,62,84^	CBCT appearance^33^
Antemortem	Present, the pulp may be sealed off naturally, and secondary dentin may form	Rounded, possibly discoloured edges. Bone callus formation if the jaw is involved	May show periapical radiolucency, root resorption, or other vital responses	Trauma-induced antemortem cracks originate on the outer enamel surface and extend inward toward the dentino-enamel junction (DEJ)
Perimortem	Absent, may have blood staining or internal haemorrhage (if bone/jaw is involved), showing trauma happened while circulation was still active. Histological analysis is suggested, but the absence of healing is key	Sharp edges, fresh break	No signs—fracture only	Trauma-induced perimortem cracks originate on the outer enamel surface and extend inward toward the dentino-enamel junction (DEJ)
Postmortem	Absent, also no haemorrhage, and no biological response	Dry, brittle, lighter, jagged break	No signs—fracture only	Heat-induced postmortem cracks originate within the dentin, progress through the dentino-enamel junction, and finally reach the enamel

Untreated caries lesions were not widely seen in the dentitions of this collection. In East and West Germany, caries levels peaked around 1970^101^ but have declined significantly over the past few decades across all age groups. In the 1990s, the caries decline was attributed to increased fluoride availability, improved dental care, and changes in oral health behaviour and nutrition^102^. Recent studies showed that, in children, the mean caries experience decreased from 1.7 in 1997 to 0.5 in 2014 for 12-year-olds^103^and from 2.89 in 1994 to 1.73 in 2016 for 6- to 7-year-olds^104^. Adults and seniors have also experienced reductions in caries, primarily due to fewer missing teeth^103^. Despite these improvements, regional variations persist, and there remains a high proportion of untreated dental decay, particularly in the primary dentition^104^.

Like the findings of dental fracture, tooth wear affected the enamel-dentine mainly, especially the anterior teeth. Research shows that anterior teeth are more affected by tooth wear compared to posterior teeth. A study of Mexican American and European American adults found significantly higher mean wear scores in anterior teeth (1.85) versus posterior teeth (1.17)^105^. Anterior teeth are most often affected by moderate to severe wear, making them suitable index teeth for assessing overall tooth wear in population surveys^106^. Multiple risk factors contribute to tooth wear, including age, gender, bite force, parafunctional habits like teeth clenching or grinding, and dietary factors such as the number of daily meals/snacks^105^. More factors include oral habits like betel nut chewing, gastroesophageal reflux disease, and abrasive oral hygiene practices^107^. These findings highlight the complex aetiology of tooth wear and its predominance in anterior dentition. Also, the high number of missing posterior teeth in this study could have contributed to this disparity in comparison.

In this study, alveolar bone dehiscences were more present than fenestrations. Both are common findings in human skulls, with prevalence rates varying across studies. Studies on modern American skulls showed dehiscences in 40.4% and fenestrations in 61.6%^108^ while Caucasian skulls from a different study reported higher rates of 53.6% and 69.6%, respectively^109^. A higher prevalence of both was seen in skeletal Class III malocclusions (lower teeth and jaw project forward compared to the upper teeth and jaw), with 61.57% dehiscences and 31.93% fenestrations^110^. Generally, fenestrations are more common in the maxilla, while dehiscences are more prevalent in the mandible. Factors associated with these defects include thin alveolar bone, tooth inclination, and possibly ethnicity and sex^108,109,111^.

The generalizability of findings is constrained, since a small sample is less likely to capture the variability of the broader population. Furthermore, when a sample is drawn from a specific origin—such as a single geographic region or cultural group—it can introduce bias because the characteristics of that sample may not represent the larger population. As a result, conclusions drawn should be considered preliminary and interpreted with caution until validated with larger, more representative samples or from a different origin. Finally, external validation of didactic representations involves testing their applicability and effectiveness across diverse populations or samples beyond the original study context. This process ensures that the representations are not limited by contextual biases and can reliably support learning in varied educational settings. By confirming consistency of outcomes across groups, external validation strengthens the generalizability, robustness, and credibility of the didactic framework. The absence of validated forensic indicators for determining the precise timing of *perimortem* fractures remains a well-recognised limitation. Despite repeated acknowledgement in the literature, no reliable morphological, histological, or biochemical markers have been established to distinguish *perimortem* trauma and tooth loss with temporal accuracy, underscoring the continued need for caution in interpretation and the development of more robust methods.

## Conclusion

In this collection, most loss of tooth substance was either caused by wearing or physical trauma that reached the dentine. Teeth missing *postmortem* were few compared to the tooth loss during life. The observable stages of the presence or absence of alveolar socket healing offer valuable criteria to enable the differentiation between *antemortem*, *perimortem*, and *postmortem* tooth loss. Understanding these sequential changes not only aids in reconstructing timelines of injury or trauma but also enhances the accuracy of forensic identification. While fracture propagation patterns and tissue changes provide preliminary diagnostic criteria for deciding tooth fracture timing, these indicators lack scientific validation for forensic applications. The assessment of *perimortem* tooth loss and *perimortem* fractures continues to challenge forensic dentists because distinguishing them from *postmortem* changes is often complicated by the circumstantial events and taphonomic processes. Continued integration of visual assessment with clinical, radiographic, and histological markers will further improve the reliability of determining post-extraction intervals. It is expected that the summary of alveolar socket changes created in this study could be used as a communication tool in legal reports and teaching.

## Data Availability

The data that support the findings of this study are available from the corresponding author upon reasonable request.
